# Clinical, CBCT and Histological Analysis of a Florid Cemento-Osseous Dysplasia with Co-Occurrence of Simple Bone Cyst in the Mandible: A Case Report

**DOI:** 10.30476/dentjods.2024.101163.2276

**Published:** 2024-09-01

**Authors:** Antoine Berberi

**Affiliations:** 1 Director Research Center, Faculty of Dental Medicine, Lebanon University, Beirut, Lebanon

**Keywords:** Cement-osseous dysplasia, Simple bone cyst, Lesion, Mandible, Maxilla

## Abstract

Cemento-osseous dysplasia (COD) is classified, by the World Health Organization as a benign fibro-osseous lesion related to the tooth and periapical area of the jaws and is considered as a benign reactive process appearing from the apical periodontium in close relation with the apices of teeth. Usually, it is asymptomatic, discovered accidentally, and affecting particularly middle-aged African women. There are four subtypes distinguished of the lesion: periapical (PCOD), focal (FCOD), florid (FLCOD) and familial florid cemento-osseous dysplasia (FFLCOD). Pseudocysts found in the jaws go by various names, including solitary bone cyst, traumatic bone cyst, or simple bone cyst (SBC). These two pathologies have been reported separately; however, their co-occurrence remains rare and the first case of FLCOD with co-occurrence of SBC was reported by Melrose *et al*. in 1976 and later a few cases been reported in the literature. The aim of this report is to describe a case of a 46-year-old oriental female diagnosed with FLCOD with co-occurrence of SBC. Under local analgesia, a surgical exploration of the cyst was performed. In addition, a biopsy with a trephine was done in the region of missing right first mandibular molar. Based on the patient clinical, radiographic, and histological findings, a diagnosis of FLCOD was made in co-occurrence with a mandibular SBC. An examination of another female family member unveils a distinctive case, and the familial factor has been ruled out. No further treatment was planned and only follow-up was suggested.

## Introduction

Cemento-osseous dysplasia (COD) is classified, by the world health organization in 2022, as a benign fibro-osseous lesion related to the tooth and periapical area of the jaws [ 1 ]. 

COD is considered as a benign reactive process appearing from the apical periodontium in close relation with the apices of teeth with a cellular similarity to the cementum [ 2 ]. COD is categorized by the substitute of mature bone with cementum or immature woven bone surrounded by moderately cellular fibrous connective stroma and avascular cementoid tissue [ 2 - 3 ].

Some factors that may contribute to its development include genetic predisposition, hormonal influences, and chronic inflammation [ 4 - 5 ]. CODs are generally symptomless, the neighboring teeth are vital, the covering gingiva is unaltered [ 6 ], and discovered accidentally when radiographs are taken for other reasons. It is predominant in particularly middle-aged African women [ 7 ]. However, when the oral flora reaches the lesion, infection can be observed and several published papers have described cases of infected COD from pulpal disease and periodontitis [ 6 , 8 - 9 ].

Inappropriate therapeutic methodologies like biopsi-es, teeth extractions, incomplete endodontic treatment, or lesion excision can lead to a secondary infection [ 10 - 11 ]. 

Moreover, complications could be related to alveolar atrophy observed under removable prosthesis [ 8 , 10 ]. Secondary infection in COD is an indication for surgical intervention, which may reduce the disease progression [ 12 - 13 ].

When CODs lesions reach to a considerable size so that the mucosa coverage on the sclerotic bone is interrupted, the vulnerability for infection is very elevated and could lead to chronic osteomyelitis [ 10 , 14 - 15 ].

Symptoms play a principal role in planning treatment, and surgeries are recommended only in symptomatic cases or in the secondary infection [ 16 - 17 ]. 

Benaessa *et al*. [ 10 ], in a retrospective study of 133 cases of COD, discovered that infection was represented in 74.7% of cases and was more observed in the florid type. In addition, osteomyelitis related to COD represented 21.8 %, and association of COD with simple bone cyst (SBC) denoted 5.3% of cases.

The incidence of osteomyelitis exhibiting as a complication of COD varies in different studies. Melrose *et al*. [ 18 ], Kawai *et al*. [ 19 ], Alsufyani and Lam [ 4 ], Owosho *et al*. [ 20 ] and Netto *et al*. [ 11 ] reported the incidence of osteomyelitis secondary to COD as 5.9%, 14.8%, 11.3%, 5.7%, and 4% respectively.

There are four subtypes of COD distinguished by the localization and type of the lesion [ 1 ]: 

(1) Periapical cemento osseous dysplasia (PCOD), which involves the apical region of the mandibular incisive [ 21 ], (2) Focal cemento osseous dysplasia (FCOD), which is usually localized in the posterior part of the mandible but could be found in other area of the jaws [ 22 ], (3) Florid cemento osseous dysplasia (FLCOD) that is more widespread and could affect more than one quadrant in the mandible and sometimes the maxilla, but the mandible is more common with a predilection for adulthood females of African origin [ 7 , 23 ], and (4) Familial florid cemento-osseous dysplasia (FFLCOD), which typically initiates earlier than the florid variant. Genetic analysis has identified the involvement of the ANO5 gene [ 24 ].

In 1976 and for the first time, Melrose *et al*. [ 18 ] defined FCOD as a group of fibro-osseous (cemental) lesions that implicate several sides of jawbones [ 25 - 27 ].

The pseudocysts found in the jaws are identified under many names such as solitary bone cyst or traumatic bone cyst or SBC [ 28 ]. 

The first case was reported by Lucas and Blum in 1929 [ 29 ] as an empty lesion without epithelial lining and without infectious etiology. Radiographically, SBCs are characterized by a well-limited radiolucent entity with cortical borders and scalloping the roots of vital teeth without affecting the periodontal ligament or the lamina dura [ 30 - 31 ]. 

These two pathologies have been reported separately; however, their co-occurrence remains rare and the first case of co-occurrence was reported by Melrose *et al*. in 1976 [ 18 ]. Few cases have been reported in the literature (Tables 1-2).

**Table 1:** Listing of publications of florid cemento-osseous dysplasia and simple bone cyst in relation with: age, gender, site, ethnic and symptoms

Clinical case report of florid cemento-osseous dysplasia (FCOD) with co-occurrence of simple bone cyst in the literature									
	Author	Year	Number Solitary Bone Cyst	Gender	Age	Type	Site	Ethnic	Symptoms
1	Melrose et al. [ 18 ]	1976	17 (14 patients)	13W/1M	42	FLCOD	16 Mand Post & 1 Maxi	13 African & 1 Oriental	ASYM
2	Miyauchi et al. [ 44 ]	1995	1	W	40	FLCOD	Mand Post	Japanese	SYMP
3	Wakasa et al. [ 45 ]	2002	1	W	34	FLCOD	Mand Post	Not Declared	SYMP
4	Mahomed et al. [ 42 ]	2005	7	1M/6W	42/43	3 COD /4 FLCOD	Mand Post	African	FLCOD= 3 SYMP / 1 ASYMP
5	Zillo Martini et al.[ 43 ]	2010	2	W	40	FLCOD	Mand Post	African	ASYM
6	Rao et al. [ 46 ]	2011	1	W	41	FLCOD	Mand Post	African	SYMP
7	Fernandes et al. [ 49 ]	2016	1	W	27	FLCOD	Mand Post	African	ASYM
8	Kojima et al. [ 47 ]	2020	1	W	39	FLCOD	Mand Post	Japanese	ASYM
9	Lee et al.[ 50 ]	2020	1	W	48	FLCOD	Mand Post	Chinese	SYMP
10	Decolibus et al. [ 7 ]	2023	85(191 patients)	82W/3M	59/59	63PCOD/43 FCOD/ 85 FLCOD	59: Mand / 23: Mand & Max / 3: Max	70 African/ 10 Caucasian/ 3 Hispanic/ 2 Asian	ASYM
11	Hajjami et al. [ 48 ]	2023	1	W	31	FLCOD	Mand Post	African	SYMP
12	Berberi A	2024	1	W	46	FLCOD	Mand Post & Ant & Max	Oriental	ASYM

Abbreviations: W= women, M= men; COD= cemento osseous dysplasia, FLCOD= florid cemento-osseous dysplasia, FCOD= focal osseous dysplasia, PCOD= periapical cemento osseous dysplasia; Mand= mandible, Max= maxilla, Post= posterior, Ant= anterior, ASYP= asymptomatic, SYMP= symptomatic

**Table 2:** Listing of publications of cemento-osseous dysplasia and focal cemento-osseous dysplasia and simple bone cyst in relation with: age, site, gender, ethnic, and symptoms

Clinical case report of COD and Focal COD with Co-Occurrence of Simple Bone Cyst in the literature									
	Author	Year	Number Solitary Bone Cyst	Gender	Age	Type	Site	Ethnic	Symptoms
1	Higuchi *et al*. [ 53 ]	1988	4	W	41	COD	Mandible: 3 Posterior and 1 Anterior	Not Declared	ASYM
2	Mupparapu *et al*. [ 41 ]	2005	1	W	41	FCOD	Mandible Posterior	African	ASYM
3	Chadwick *et al*. [ 52 ]	2011	23	20W/3M	42/47	COD	22 Mandible / 1 Maxilla	Not Declared	ASYM

Abbreviations: W= women, M= men; COD= cemento osseous dysplasia, FCOD= focal osseous dysplasia, ASYP= asymptomatic

The aim of this report is to describe a case of a patient diagnosed with FLCOD with co-occurrence of SBC in the mandible based on the clinical, radiographical, and histological findings. 

## Case Presentation

A 46-year-old oriental female was referred to our clinic, with a panoramic radiograph, complaining of an uncomfortable sensation on the posterior part of the left mandible.

Patient’s medical history was clear with no evidence of systemic disease or mandibular trauma. Extraoral examination revealed no abnormal symptoms or adenopathy. Intra oral examination showed buccal bone expansion in the left mandibular third molar area and a missing second mandibular left premolar and first right mandibular molar.

A history of extraction of the second mandibular left premolar during the childhood and the first and third mandibular right molar, as well as the third maxillary right molar was mentioned and dated ten years ago.

**Figure 1:**
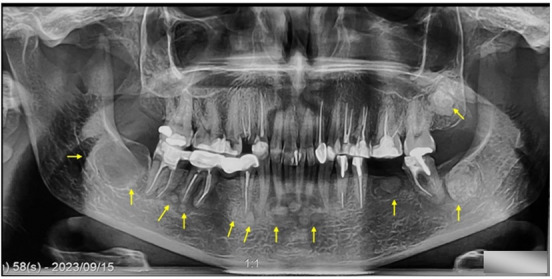
Panoramic radiograph showing the cystic lesion with the cemento-osseous dysplasia lesions in the mandible and maxilla

Panoramic radiograph revealed a single well-defined radiolucent cyst-like lesion situated in the location of the missing left third mandibular molar with an expansion to the distal root of the second left molar, which linked to the clinical symptoms (Figure 1).

Radiopaque lesions bordered by radiolucent halo are noted in relation with the apices of endodontic treated first and second mandibular left molars, and first left mandibular premolar without any clinical signs. 

A periapical mixed radiopaque-radiolucent lesions were located on the apices of the vital mandibular canines and incisors teeth, also, the lamina dura surrounding the apical areas of the mentioned teeth was absent. In addition, radiopaque lesions surrounded with a thin layer of radiolucent area present in the region of extracted first and third right mandibular molars and the third maxillary right third molar. 

For a better planning and delimitation of the lesions, a cone beam computed tomography (CBCT) was prescribed. 

**Figure 2:**
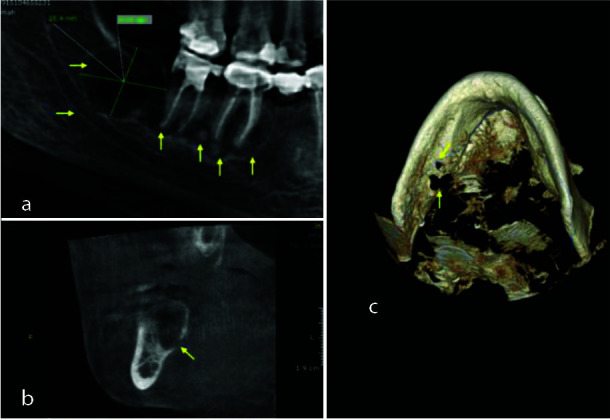
**a:** panoramic view of the cone beam computed tomography showing the cystic lesion measurement and cemento-osseous dysplasia lesions related to the apices of the teeth, **b:** Paraxial image of the cystic lesion revealing the lingual bone perforation, **c:** A 3 D reconstruction displaying the lingual bone perforation

Regarding the single well-defined radiolucent cyst like lesion in the left third mandibular molar, the CBCT revealed a well-defined expansile osteolytic lesion measuring approximately 18.4 x 14.9 mm in size in the left body of the mandible in continuity of the distal root of the second molar, above the mandibular canal with an expansion of the buccal cortical bone and a discontinuity of the lingual cortical was noted (Figures 2a-c).

Based on these radiological findings, a differential diagnosis of the radiolucency lesion was in favor of residual cyst, unicystic ameloblastoma, odontogenic keratocyst, and SBC.

**Figure 3:**
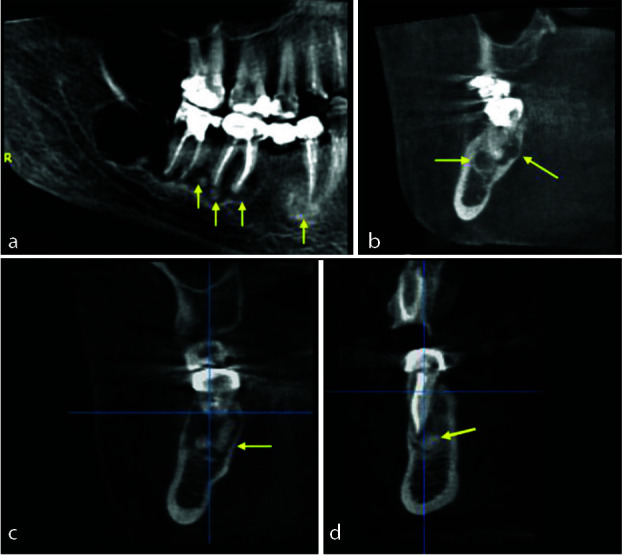
**a:** Panoramic reconstruction exhibiting the periapical lesions located in apices of the left mandibular, **b:** Paraxial viewing the lesions associated with the second molar, **c:** Paraxial presenting the lesions associated with the first molar, **d:** Paraxial exposing the lesions associated with the first premolar

Regarding the periapical lesions located in apices of the left mandibular molars and premolar, the paraxial images showed a radiopaque image surrounded by a radiolucency image in relation with the apices of the molars and premolar (Figures 3a-d).

The panoramic reconstructed CBCT images revealed a periapical mixed radiopaque-radiolucent lesions located in the anterior part of the mandible. 

**Figure 4:**
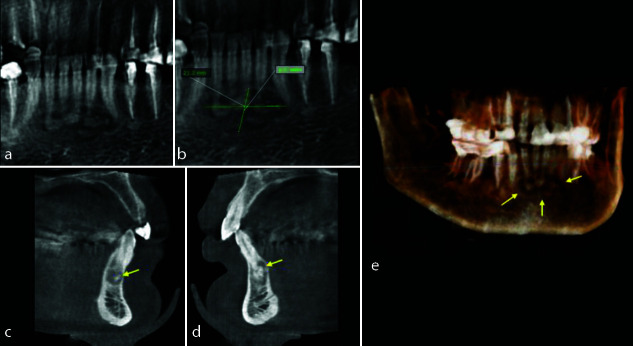
**a:** Panoramic reconstruction of the anterior mandible revealing the mixed radiopaque-radiolucent lesions, **b:** Measurement of the extension of the lesion, **c:** Paraxial cut of the canine, **d:** paraxial cut of the lateral incisive, **e:** 3D reconstruction showing the buccal extension of the lesions

The lesion extended from the mesial side of the left mandibular lateral incisor to the mesial side of the right mandibular canine, appearing radiopaque with a radiolucent rim. It seems that in this multifocal lesion, solitary lesions coalesced to form a larger lesion. The total dimension of the lesion measured about 21.2 mm in the mesiodistal direction and 11.5 mm in the longest superior-inferior direction. The paraxial images demonstrated a continuity of the buccal and lingual cortical bone. (Figures 4a-e) As all the concerned teeth were vital and there were no clinical symptoms or discomfort and based on the radiographic aspects of the lesion, the diagnosis of PCOD was established.

**Figure 5:**
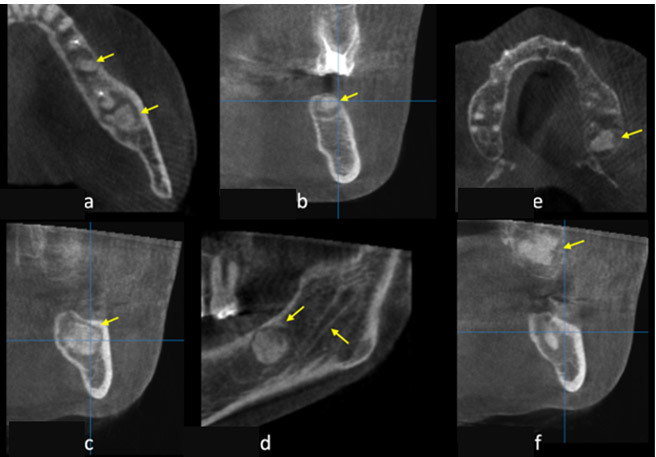
**a:** Axial image of the mandible showing the localization of the two lesions, **b:** Paraxial image of the first molar lesion, **c:** Paraxial image of the third molar lesion, **d:** Sagittal image of the third molar lesion above the mandibular canal, **e:** Axial image of the maxillary lesion, **f:** Paraxial image of the maxillary lesion

The radiopaque lesion with a radiolucent halo, situated in the right mandible and maxilla, associated with previous extractions, was examined through paraxial images. These images revealed a well-defined lesion within the mandibular and maxillary bones (Figures 5a-f).

The differential diagnosis was ossifying fibroma or idiopathic osteosclerosis or condensing osteitis or cementoblastoma.

**Figure 6:**
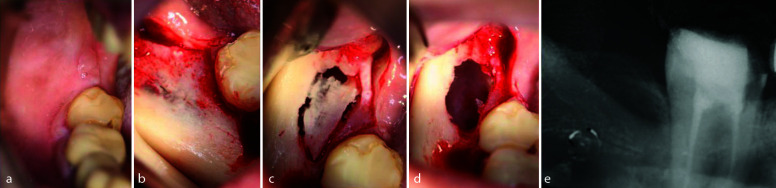
**a:** Preoperative picture, **b:** Buccal bone exposition, **c:** Buccal osteotomy, **d:** Empty cavity in relation with simple bone cyst, **e:** Post-operative intra-oral radiograph at two months

After obtaining consent from the patient and under local analgesia, a surgical exploration of the cyst at the left angle was performed. Intraoperatively, the cyst revealed an empty cavity in the bone with no lining epithelium, leading to the diagnosis of the SBC (Figures 6a-e).

**Figure 7:**
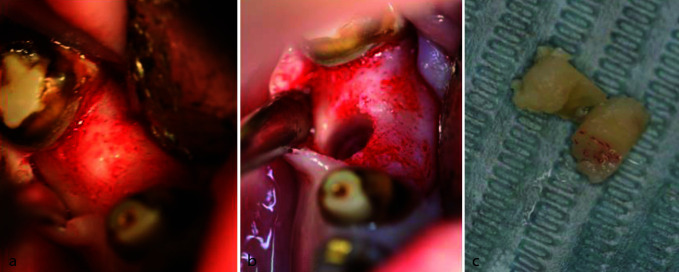
**a:** Bone exposition, **b:** Drilling with the trephine, **c:** Biopsy material

In addition, a biopsy with a trephine bur was done in the region of missing right first mandibular molar (Figures 7-c).

**Figure 8:**
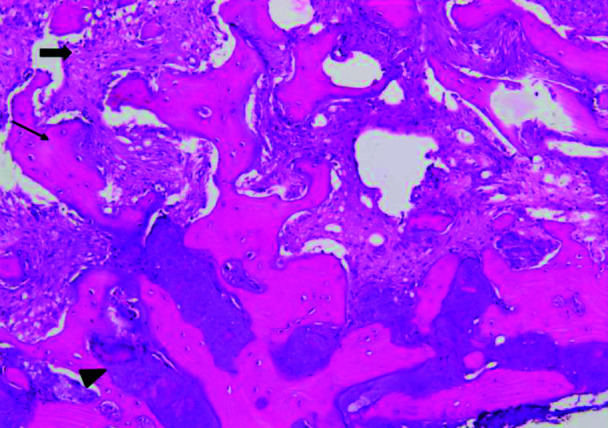
The lesion showed woven bone trabeculae and cementum-like mineralization within cellular fibrous connective tissue (Hematoxylin-Eosin 200 x)

The histology images displayed a trabeculae of woven bone surrounded by fibrous connective tissue with a deposit of cementum like mineralization. The diagnosis was in favor of COD (Figure 8).

Based on the patient clinical, radiographic, and histological findings, a diagnosis of FCOD was made in cooccurrence of a mandibular SBC. 

Further investigation into the other female family members showed a unique case and the familial factor was excluded. No further treatment was planned and only clinical and radiological follow up was proposed.

## Discussion

COD is a pathologic condition reported with a female predilection (82.9–94.3%) and preference for females of African descent [ 4 , 7 , 22 - 23 ].

Differential diagnoses of COD are sclerosing osteomyelitis (SO), Gardner’s syndrome, fibrous dysplasia, multiple cemento-ossifying fibroma (COF), Paget’s disease, and gigantiform cementoma [ 2 ].

COD usually appears in both sides as multiple asymptomatic masses whereas SO is mostly observed in one side with no pain history or infection or trauma and no radiolucent margins were described [ 32 ]. SO appears as a single, poorly delineated opaque segment of the mandible, whereas COD is seen as multiple round or lobulated opaque masses [ 32 - 33 ].

COD does not involve skin lesions, skeletal expressions, or any related dental anomalies like in Gardner’s syndrome [ 28 ].

When extensive lesions are observed in all four quadrants of the jaws, COD could be confused with fibrous dysplasia and the difference is based on irregular sclerotic lesions observed in COD whereas fibrous dysplasia displays uniform ground glass appearance incorporate in healthy bone [ 2 ].

COD was seen predominantly in black women whereas COF showed no female predilection and COF occurred in patients with an average of 10 years younger than patients with COD. Most patients with COD were asymptomatic and patients with COF displayed jaw expansion and a considerably larger size lesion [ 2 , 34 - 35 ]. Radiographically, cases of COD mostly demonstrated an irregularly mixed radio-opacity, whereas COF presented as radiolucent lesion; moreover, COF usually exhibit more buccolingual growth than COD [ 34 - 35 ].

Paget’s disease is distinguished by a cotton-wool appearance that touches the entire mandibular bone and exhibits loss of lamina dura, while COD is localized above the mandibular canal [ 2 ].

Gigantiform cementoma is usually seen most frequently in young people [ 36 - 37 ]. Radiographically, it typically presents as multi-quadrant, expansile, mixed radiolucent-opaque lesions that cross the midlines of the jaws, showing considerable, diffuse, and disfiguring expansion early in the disease process and are not fused to the tooth root [ 24 , 36 - 37 ].

Decolibus *et al*. [ 7 ] stated that FLCOD represent 44.5% of COD. However, other studies reported that the highest shared types of COD are both PCOD and FCOD [ 3 , 21 , 23 ]. 

The most frequent subgroup was reported to be PC-OD [ 1 , 7 ] but Pereira *et al*. [ 23 ] reported that FLCOD was the most common form and for Günaçar *et al*. [ 35 ] the most common types were FCOD and FLCOD. 

PCOD and FCOD are often asymptomatic, typically discovered incidentally during routine radiographic analysis. This observation aligns with findings reported by Alsufyani and Lam [ 4 ] (72.2%), Decolibus *et al*. [ 7 ] (85.3%), and Owosho *et al*. [ 20 ] (77.1%).

Pain and infection are reported with the FLCOD and FFLCOD types [ 17 , 24 , 38 ].

The mandible is the most frequently affected jawbone by COD lesions, accounting for 90% of such cases. These lesions are typically associated with the apices of mandibular teeth and are positioned above the lower alveolar canal [ 4 , 7 ].

In our case, all the lesions were located in the apices of endodontic treated teeth for the posterior part in the mandible and in relation with vital teeth in the anterior part of the mandible and no relation with mandibular canal were detected. The periapical type displays a tendency for the anterior part of the mandible while the focal and florid types are present in the posterior area of the mandible [ 2 , 21 ]. 

SBCs are more common found in the mandible than they are in the maxilla [ 30 , 31 ] and more frequently in male than in female, with a 3:2 ratio [ 31 ]. 

These lesions are more frequently detected in the mandibular bone and localized above the inferior mandibular canal, and may appear in the incisor area of the mandible [ 30 , 39 ].

The etiopathogenesis of SBC remains uncertain. The most widely acknowledged theory is the traumatichemorrhagic hypothesis, with reported incidence rates ranging from 17% to 70% in different case series [ 31 , 40 ]. 

Mupparapu *et al*. [ 41 ] stated that the etiology of SBC is a venous obstruction and blockage of interstitial fluid drainage, in the remodeling cancellous bone area.

The treatment involves a simple procedure consisting of a deep curettage of the bony walls to provoke bleeding which in turn leads to the production of a new bone that usually takes 6–12 months for a complete bone healing [ 30 - 31 , 40 ].

FLCOD has been reported in the literature to co-occur with SBC, and it appears to manifest more frequently in the elderly population, as observed in a case series:

Melrose *et al*. [ 18 ] reported a case series of 34 patients with FLCOD, 14 patients had presented 17 SBC (16 in the mandible and 1 in the maxilla) in co-occurrence of SBC. Mahomed *et al*. [ 42 ] reported seven cases, three cases of COD and four of FLCOD associated with SBCs. Decolibus *et al*. [ 7 ] described a clinical analysis of 191 cases of COD, in which 85 patients were diagnosed with FLCOD. Additionally, Zillo Martini *et al*. [ 43 ] reported two cases of FLCOD associated with SBC. 

In addition, case reports of FLCOD in association with SBC in the mandible with single SBC [ 44 - 48 ] or multiple SBCs [ 49 , 50 ] have been reported. 

SBC in co-occurrence with the FLCOD has a female predilection and is mostly observed in middle-aged African and Asian women (40–50 years) [ 7 , 18 , 42 - 50 ] as in our case, the patient was oriental with 46 years of age and presented with a single SBC. 

The definite female predilection may provide clues to the etiology, as hormonal discrepancies disturbing bone metabolism have been proposed as a causative element [ 23 , 42 ].

Günaçar *et al*. [ 35 ] reported that the most affected area in the florid group is the mandible, particularly the anterior region and rarely the maxilla and usually two or more quadrants of the mandible are involved [ 7 , 22 , 42 ]. Our patient presented involvement in three mandibular quadrants and one maxillary. 

SBCs are uncommon in elderly, when they appear; they are usually linked with a fibro-osseous lesion. Horner *et al*. [ 51 ] and Mahomed *et al*. [ 42 ] and Chadwick *et al*. [ 52 ] have mentioned that SBC in the elderly patient present different etiopathogenetic differences from SBC that appears in young individuals. 

In the younger population, the disruption of osteoclastic-osteoblastic activities may be linked to the ongoing changes in the biomechanical properties of the mandible during development.

In mature individuals, particularly potentially osteoporotic women, COD-associated SBCs are considerably more common, which may be attributed to low or inadequate osteoblast numbers [ 52 - 53 ].

It is thought-provoking to understand why there is co-occurrence of florid cemento-osseous dysplasia with simple bone cyst.

The histological and radiological features of FLCO-D may be related with the developmental stage of the lesion [ 2 , 4 , 21 - 22 , 18 , 35 , 41 , 52 ]: 

In the early stage (osteolytic stage), the lesion is predominantly radiolucent and composed of cellular fibrous tissue with scattered foci of mineralization which is the result of the lamina dura and periodontal.

In the intermediate stage (cementoblastic stage), the lesion is mixed radiolucent and radiopaque and composed of fibrous tissue with irregular trabeculae of cementum-like material.

In the mature stage, the lesion is predominantly radiopaque and frequently surrounded by a radiolucent halo; it composed of dense cementum-like material with minimal fibrous tissue. 

The radiological findings of our patient revealed that the posterior mandibular and maxillary lesions are in a mature stage, but the anterior mandibular lesions are in cementoblastic stage.

The histological outcomes were in favor of the mature stage.

As the lesion matured, there was a progression of the radiopaque mass accompanied by a decrease in blood vascularization. Simultaneously, there was an obstruction of lymphatic drainage within the fibrous connective tissue surrounding the cystic cavity. This observation suggests that the disorderly bone production in FLCOD could potentially result in cystic degeneration, as noted by previous studies [ 44 , 46 , 52 ]. Melrose *et al*. [ 18 ] also supported a similar etiological hypothesis, reinforcing the notion that the cystic appearance follows the development of FLCOD.

Miyauchi *et al*. [ 44 ] noted the presence of a prominent capillary network within the proliferating fibrous tissue and throughout the medullary spaces between the newly formed hard tissue trabeculae around the cystic cavity, designating a potential for cystic lesion formation after the development of COD. Wakasa *et al*. [ 45 ] described a case in which SBC was located in the same area where florid COD was three years before, and they suggest that florid COD heads SBC formation. 

We cannot be sure if the SBC was located in previous lesion due to lack of previous data in our case however, we can state that the lesions are observed in the apices of endodontic treated teeth in the mandible but not in the maxilla. This discrepancy could be associated with the differing vascularization patterns in both jaws.

The existence of lesions, notably in the posterior left mandible and maxilla within the areas of previous extraction, radiologically characterized by a radiopaque core enveloped by a radiolucent halo and histologically composed of dense cementum-like material with minimal fibrous tissue, might be indicative of a disruption in osteoclast-osteoblast activities.

Regarding the site of the second mandibular left premolar extracted during the childhood, no lesion was detected, and the most probable reason could be that the extraction and the alveolar healing preceded the hormonal perturbations.

## Conclusion

Based on clinical, radiological, and histological findings, we present an intriguing case of FLCOD associated with SBC, manifesting in four-quadrant locations, three in the mandible and one in the maxilla. The lesion types varied, correlating with the apices of endodontically treated teeth in the posterior mandible, apices of vital teeth in the anterior mandible, and extraction sites in the posterior mandible and maxilla. Analyzing the data, we hypothesize that the concurrent presence of FLCOD and SBC may be attributed to disruptions in osteoblast-osteoclast activities. Following the assignment of the diagnosis, we recommend a clinical and radiological follow-up exclusively.
